# Tracking the Interplay between Bound Peptide and the Lid Domain of DnaK, Using Molecular Dynamics

**DOI:** 10.3390/ijms140612675

**Published:** 2013-06-17

**Authors:** Itzhaq Azoulay, Nataly Kucherenko, Esther Nachliel, Menachem Gutman, Abdussalam Azem, Yossi Tsfadia

**Affiliations:** Department of Biochemistry and Molecular Biology, George S. Wise Faculty of Life Sciences, Tel Aviv University, Tel Aviv 69978, Israel; E-Mails: itzik194@gmail.com (I.A.); nataly.kucherenko@gmail.com (N.K.); eti@hemi.tau.ac.il (E.N.); me@hemi.tau.ac.il (M.G.); azema@tauex.tau.ac.il (A.A.)

**Keywords:** Hsp 70 chaperone, DnaK, reaction mechanism, molecular dynamics

## Abstract

Hsp70 chaperones consist of two functional domains: the 44 kDa Nucleotide Binding Domain (NBD), that binds and hydrolyses ATP, and the 26 kDa Substrate Binding Domain (SBD), which binds unfolded proteins and reactivates them, utilizing energy obtained from nucleotide hydrolysis. The structure of the SBD of the bacterial Hsp70, DnaK, consists of two sub-domains: A β-sandwich part containing the hydrophobic cavity to which the hepta-peptide NRLLLTG (NR) is bound, and a segment made of 5 α-helices, called the “lid” that caps the top of the β-sandwich domain. In the present study we used the *Escherichia coli* Hsp70, DnaK, as a model for Hsp70 proteins, focusing on its SBD domain, examining the changes in the lid conformation. We deliberately decoupled the NBD from the SBD, limiting the study to the structure of the SBD section, with an emphasis on the interaction between the charges of the peptide with the residues located in the lid. Molecular dynamics simulations of the complex revealed significant mobility within the lid structure; as the structure was released from the forces operating during the crystallization process, the two terminal helices established a contact with the positive charge at the tip of the peptide. This contact is manifested only in the presence of electrostatic attraction. The observed internal motions within the lid provide a molecular role for the function of this sub-domain during the reaction cycle of Hsp 70 chaperones.

## 1. Introduction

Molecular chaperones of the 70 kDa Heat Shock Protein (Hsp70) family belong to a highly conserved, ubiquitous family of proteins found in most of the cell’s compartments in all three kingdoms of life—Archaea, Bacteria and Eukarya [1–4]. Hsp70 proteins participate in various cellular functions, such as folding of newly synthesized proteins, preventing protein aggregation and targeting proteins to degradation or translocation across membranes [5–8]. Hsp70 proteins also share the ability to recognize denatured proteins by their exposed hydrophobic moieties that interact with the Substrate Binding Domain (SBD) core.

**Figure 1:**
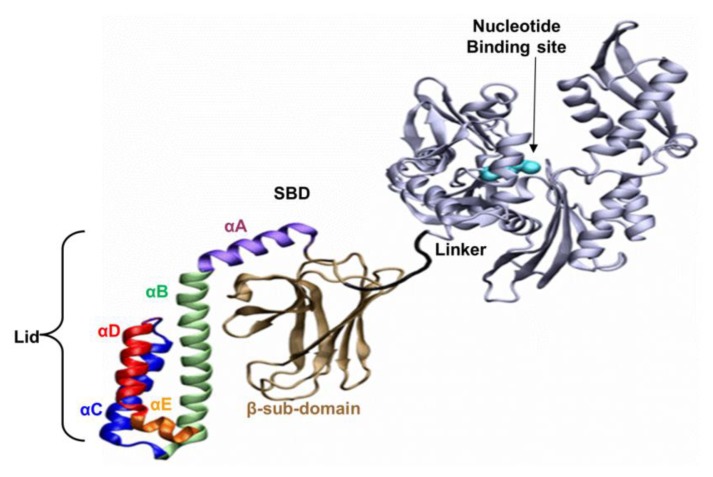
Hsp70 structure. The two functional domains of DnaK: the nucleotide binding domain—NBD (grey), joined to the substrate binding domain—SBD through a linker (black). A peptide substrate can be bound to the cavity in the β-structure and near the lid, whose five different segments are color coded. The presented diagram is missing the 35 residues on the C terminal section that were not resolved by crystallization (PDB ID: 2KHO).

The well-known bacterial homologue of the family, DnaK, which serves as the model system in this study, is composed of 638 amino acids. Its total molecular weight is approximately 70kDa, and it has two functional domains: (i) a ~44 kDa regulatory Nucleotide Binding Domain (NBD), which is responsible for the binding and hydrolysis of ATP; and (ii) a ~26 kDa SBD, which is responsible for the binding and folding of polypeptide chains (Figure 1). The activities of the two domains are allosterically coupled via a short (7 amino acids) flexible linker, which affects the structural changes of the SBD [1,9–13].

Based on the solved crystal structure of the DnaK-SBD (residues 389–607), it is now possible to distinguish between two sub-entities: (i) a conserved ~15 kDa β sandwich domain (residues 389–507), which consists of two sheets of four antiparallel β stands, (β1–8) that form a hydrophobic cavity which serves as a substrate binding site; (ii) a less conserved α helical domain (residues 508–607) of five segments (αA–E), also known as the “Lid”, which is suggested to control the accessibility of the peptide to the binding cavity. The last 30 residues on the C terminal domain (numbered 608–638) were not resolved by crystallization and are suggested to assume a random coil shape. Recent studies have shown that these residues enhance chaperone activity [14,15].

The association of the protein with its substrates is controlled by adenine nucleotides. When ATP is bound to the NBD, DnaK exhibits fast reversible association/dissociation dynamics, resulting in low substrate affinity. In the ADP-bound state, the dynamics are slower and the affinity for the substrate is much higher. These altered properties are derived from the structure of the SBD: in the ATP-bound state, the entire protein has a more condensed conformation, whereas in the ADP-bound state its structure is more relaxed [16–19].

The peptide binding site of the protein is located between the two β-sheets of the SBD, which form a large hydrophobic cavity with a surface area of 1713 Å^2^[20]. Adjacent to this cavity is a five helices structure commonly referred as the “lid”. Screening of peptide libraries for sequences that are bound to the SBD revealed that binding calls for short sequence of hydrophobic peptides (~7 residues), with no anionic residues and a few positively charged amino acids, consistent with a general identification motif of ±HyHyHyHyHy± (where ± and Hy stand for positively charged and hydrophobic residues, respectively) [4,12,13,21–23]. The crystal structure of the SBD in complex with a seven-residues-long peptide; Asn^1^-Arg^2^-Leu^3^-Leu^4^-Leu^5^-Thr^6^-Gly^7^, abbreviated by NR, which was reported by Zhu *et al.* in 1996 (PDB ID: 1DKX) [20], was a milestone which made a great contribution to the understanding of the mechanism of recognition and specificity of Hsp70s. The crystal structure revealed that the three core leucine residues of the NR peptide are all well buried inside the binding pocket of DnaK, while the residues Arg^2^ and Thr^6^ are partially exposed to the solvent, leaving the Asn^1^ and the Gly^7^ entirely out of the pocket. Asn^1^ is facing the αB helix of the lid, while Gly^7^ is protruding on the other side of the peptide binding domain [20]. In accord with a proposal from Van Durme *et al.* [24], the peptide’s core should be hydrophobic with positive moieties on both ends. It was suggested that the negatively charged residues in the lid sub-domain may play an important role in the regulation of substrate binding. By designing peptides of different lengths and properties, and testing their affinity *in vitro* to Eukaryote Hsp70, Misra *et al.* [25] arrived at the same conclusion. In the present study, the role of the positive charge of the substrate, and its significance for the molecular mechanism of DnaK and Hsp70 proteins, are for the first time investigated using molecular dynamics tools.

The common model suggests that when ATP is bound to the NBD, the lid sub-domain stays in an “open” configuration detached from the β subdomain, enabling an easy access for the client peptide into the binding pocket. Upon ATP hydrolysis, the lid sub-domain shifts into a closed form, preventing binding of a new peptide or its release. It is generally assumed that the entire lid is a rigid body, which participates in the stabilization of the DnaK-peptide complex and indirectly controls the binding of the peptide. In fact, the role of the lid in the inter-domain communication is still debatable [21,22,26–28]. While some studies suggest that a complete deletion of the lid has no effect on the ATPase activity of the DnaK protein, others had suggested that mutations, or any deletions in the lid, severely damage the chaperone and hinder the growth of cells [11,29]. Moro *et al.* [30] focused their studies on the importance of the α helices of the SBD, proposing that ionic contacts between the N-terminal region of helix B of the lid and the β-sandwich subdomain are necessary to stabilize the interaction between the lid and the β-sandwich subdomain in the ADP-bound state, thus controlling the stability and functionality of the protein-substrate complex.

In the present study we implemented molecular dynamics (MD) simulations of the SBD in a complex with the NR peptide (PDB ID: 1DKX) in order to examine the changes in the conformation of the lid once the protein is relaxed from the forces operating during the crystallization process. For this purpose, we decoupled the NBD from the SBD, limiting the simulations to the structure of the SBD section. Our main interest was to understand how the positive charge on the peptide influences the internal interactions of the SBD, which complements the recent MD studies of the NBD/SBD interdomain communication in Hsp70 that were carried out with no peptide bound in the SBD domain [31,32]. The present simulations indicated that the lid is not a rigid structure and exhibits well defined structural changes, where the terminal helices of the lid reoriented themselves with respect to the bound peptide, in a mode depending on its charge. For both native and the double charged peptide (NR and N1R), helix D of the lid was attracted toward the N terminal moiety of the peptide. Upon omission of the positive charge of the peptide, the lid was less mobile and no contact between the lid and the peptide was formed. We propose that the residues on helix D may function as a hook that assists in the extraction of the peptide from its hydrophobic binding site within the β sandwich of the SBD.

## 2. Results

### 2.1. List of Simulations

The carried out simulations, were all based on the crystal structure of the DnaK SBD (PDB ID: 1DKX) and are detailed in Table 1.

Simulations 1 and 2 were aimed to check the stability of the β sub-domain. For that purpose, the helical lid was truncated from the original protein structure, and only the β-sandwich sub-domain was simulated. Each of these simulations was repeated twice in the presence of the peptide and twice in an Apo state, where the peptide was removed. In these simulations, the presence or the absence of the peptide did not affect the stability of the β sub-domain.

Simulation 3 was of the holo-complex consisting of DnaK’s SBD and the hepta-peptide as embodied in the crystal coordinates file (PDB ID: 1DKX). This simulation was repeated twice, yielding essentially the same results.

In simulation 4 the peptide was removed from the structure and the SBD protein was simulated in the absence of the peptide. This simulation was repeated twice, yielding essentially the same results.

Simulations 5 and 6 were carried out with modified peptides, modulating the charge of the peptide. In simulation 5 the first residue of the peptide was replaced by Arginine and in simulation 6 the positive charge of the peptide was eliminated by replacing the Arginine by Alanine.

Simulation 7 was carried out with mutated DnaK protein, reproducing *in-silico* the experiment of Aponte *et al.* [14] who noticed that this mutant had a lower chaperone-like activity.

The structural stability of the β sub-domain of the SBD was evaluated by calculating the RMSD of the protein in simulation 1 and 2. In both cases, the RMSD of the backbone atoms was less than 4 Å, a value attributed mostly to the motion of the loops connecting the strands. During these simulations, the distance between the centers of mass (peptide and the β sub-domain) was constant (11.38 ± 0.49 Å) and with RMSF values of 0.19 ± 0.09 Å, indicating that the peptide is tightly anchored within the β sub-domain and the complex has a very rigid structure (data not shown).

### 2.2. Simulation of the Apo and Holo Complexes

**Figure 2:**
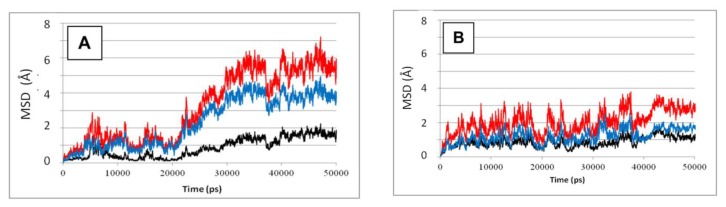
Structural stability of the SBD. The relative contribution of the different domains to the Mean Square Deviations (MSD) of the entire protein in: (**A**) SBD^NR+^ simulations; (**B**) SBD^NR−^ simulations. The MSD of the full structure is shown in red, while the β-subdomain and the lid are colored in black and blue, respectively.

Figure 2 depicts the MSD values calculated for the whole SBD (according to Equations 1–4), either in complex with a peptide (frame A) or in its absence (frame B). The difference between the two simulations is clear—the presence of the peptide definitely increases the structural fluctuation of all sub-elements of the SBD. In the absence of the peptide the total MSD stabilizes at 0.28 ± 0.04 Å for *t* ≥ 40 ns, where the relative contribution of the lid (blue) and β sub-domain (red) are almost equal. In the presence of the peptide, the total MSD for *t* ≥ 40ns is 0.55 ± 0.05 Å, and the lid contributes ~70% of that value. Apparently, the presence of the peptide initiates some internal forces that reorder the structure of the complex. This conclusion is in accord with the recent computational work published by Milanesi, Morra and coworkers [32]. In their study, both NBD and SBD sections were simulated in the absence of a bound substrate. Inspection of their trajectories revealed that the lid segment retained a constant orientation with respect to the β sub-domain, both in the presence and absence of ATP. Apparently, the relative motion of the helices with respect to the β sub-domain, reported in our study, is a consequence of the interaction of the lid helices with the bound peptide.

### 2.3. The Interaction of the Substrate Binding Domain (SBD) with the NR Peptide

#### 2.3.1. Spatiotemporal Analysis of the Lid-Peptide Interaction

**Figure 3:**
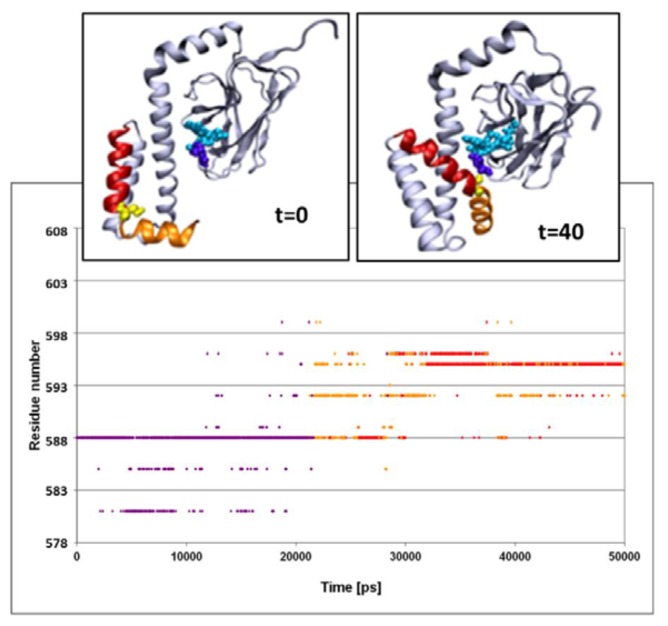
Sites of interactions of the lid with the peptide. Spatiotemporal analysis of the data from the SBD^NR+^ simulation, characterizing the interacting residues between the lid helices and the bound peptide. The *Y* axis represents the amino acid of helices D and E. Red and orange dots stand for distances smaller than 3 Å or between 3 and 6 Å, respectively. Purple indicates distances greater than 6 Å. Insets: Snapshots presenting the structure of SBD at the end of the simulation (right inset) compared to the one at the starting point (left inset). A cartoon diagram of the DnaK-SBD (grey), emphasizing helices D and E (red and orange, respectively) and Ser^595^ in CPK (yellow). The NR peptide is presented in CPK, colored in cyan, except for the Asn^1^ (purple).

The structural fluctuations experienced by the complex are shown in Figure 3, which is a spatiotemporal presentation of the interaction between the lid’s helices and the bound peptide, as calculated for the WT simulation (simulation 3 in Table 1). The *Y* axis denotes the sequential number of the lid residues, and the *X* axis denotes the time vector of the simulation [33]. The program identifies, at each timeframe, the SBD residue nearest to the peptide, and colors it in accordance with the distance; if the nearest residue was at the distance of more than 6 Å from the peptide, the dot is colored in purple. For residues between 6 and 3 Å, the color is yellow, and if the separation was less than 3 Å, the given color is red. Due to the rapidness of the structural fluctuations, the identity of the “nearest residue” alternates between those residues that are in the highest proximity to the peptide, while all others are screened out.

The main frame of Figure 3 depicts the evolution of the contact between the residues of the lid with the peptide. At the first 23 ns of the simulation, the minimal distance between any of the lid residues and the peptide exceeds 6 Å. For the next few ns several of the lid residues approach the peptide, until a constant short-distance contact is established between the NR peptide and Ser^595^.

The structural changes associated with the formation of a tight contact between Ser^595^ with the peptide are presented by the two snapshots inserted in the figure; on the left is a snapshot taken at *t* = 0 and on the right is one at *t* = 40 ns. At *t* = 0, the lid is still in its crystal state configuration, where helices D and E (red and orange, respectively) appear to have an “L” shaped structure. At this initial structure the minimal distance between Ser^595^ (colored in yellow) and Asn^1^ of the peptide is 22 Å. During the first ~20 ns of the simulation this initial distance is retained, but then a rapid structural rearrangement occurs, and the two residues come into close contact (2.31 ± 0.95 Å), which lasts until the simulation is terminated (right inset). The same structural transitions were noticed on repeating runs of the same system. In the second run the contact between the residues located at the junction between helix D and E took place much faster, reaching a stable configuration within the first 15 ns. Extension of the simulation for another 5 ns made no further changes.

#### 2.3.2. The Effect of the Peptide Charge on Its Interaction with the Lid

The NRLLLTG peptide, with which the DnaK protein was crystallized, carries a positive charge near its N terminus. As a next step in our study, we examined the possibility that electrostatic attraction affects the dynamics of the lid. For that purpose, two simulations were carried out; in one case the positive charge on the N terminus of the peptide was doubled by replacing its residue with arginine RRLLLTG, (N1R, simulation 5). In the second case, the charge of the peptide was eliminated by replacing the arginine with alanine NALLLTG, (R2A, simulation 6).

**Figure 4:**
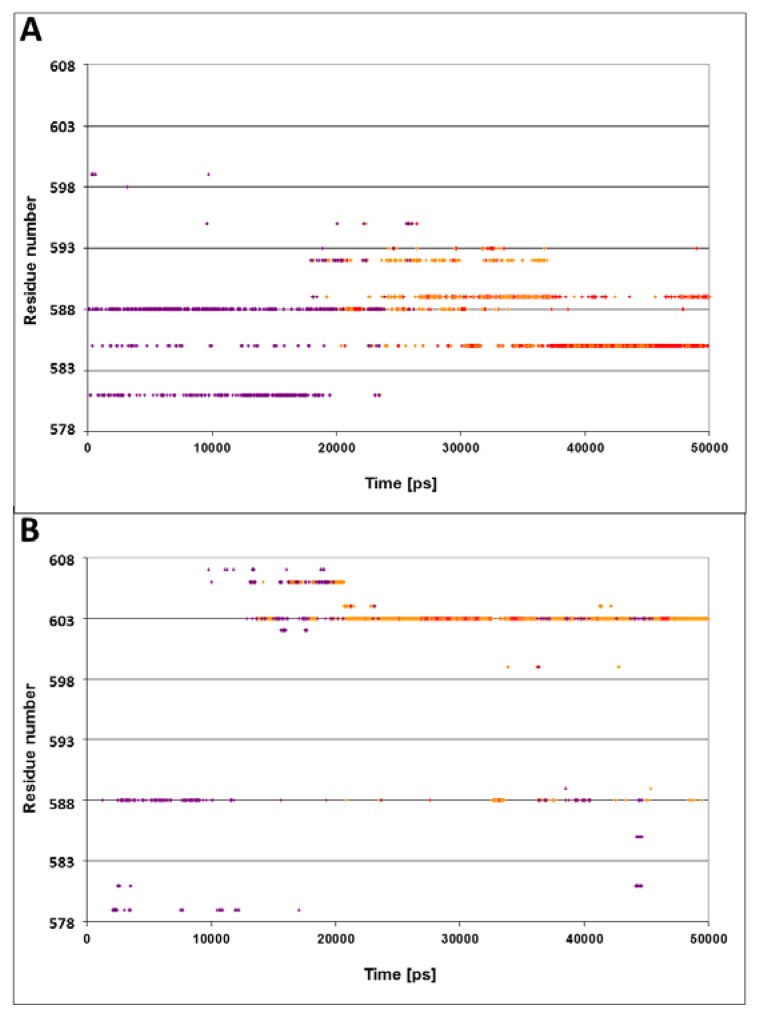
The effect of the peptide’s charge on the proximity to the residues of the lid. The dots describe the spatiotemporal analysis of the simulations of the complexes with either N1R mutation (double charged) of the peptide (panel **A**) or the uncharged mutated peptide R2A (panel **B**). The color code for the distances is as in Figure 3.

Figure 4A presents the spatiotemporal analysis of the trajectory calculated for the N1R peptide, bearing a double positive charge. The initial distances between the residues on helices D and E and the peptide are ~10–15 Å, with a gradual approach that takes ~20 ns. By the end of the simulation, helices D and E form close contacts with the N-terminus of peptide. The closest residues in this case are Gln^585^ and Gln^589^, both located in vicinity to the junction between helix D and E. These results suggest that the interaction of the lid with the positive charge of the peptide is stabilized not only by a single residue (Ser^595^), but by a number of residues in its vicinity.

The omission of the positive charge (Figure 4, panel B) markedly weakens the contact between the lid and the peptide. The only moiety that is relatively close to the peptide is Gln^603^, and even this residue does not approach close enough to form any contact, fluctuating most of the time in the range of 3–6 Å.

The conclusions from these two simulations are pretty clear: the lid was attracted to the N-terminus of the peptide by its positive charges, while, in the absence of electrostatic attraction, the lid failed to reorient and the distance between Ser^595^ and Asn^1^ remained more than 10 Å through the whole length of the simulation.

### 2.4. Strength of Interaction between Defined Residues

**Figure 5:**
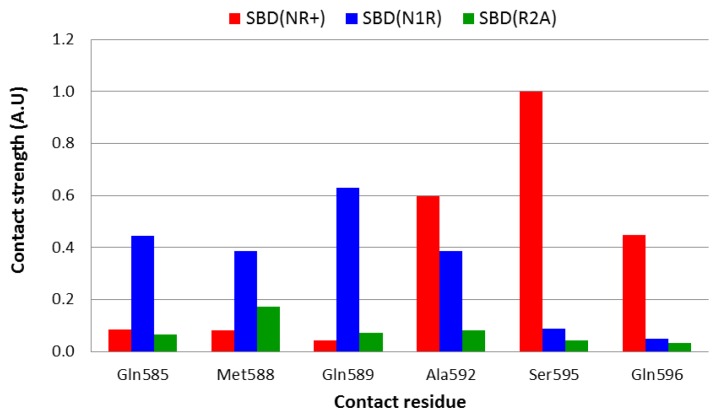
Normalized strength of interaction between the peptide and the lid, for the DnaK-SBD-Peptide simulations. The calculations were carried out using Asn^1^ (of the peptide) as the point of reference, and the strength parameter was calculated according to Equation (6). For comparative purpose the value of the strength parameter calculated for the interaction of S^595^ with Asn^1^ was defined as 1, and all others were scaled appropriately. The results represent the last 10 ns of each simulation, when the system had reached a stable conformation. The presentation is limited to only six residues which were closest to N^1^ residue of the peptide. The color code is defined in the frame.

Even in a stable, well converged structure, the distance between nearby moieties is not a stable function due to constant structural fluctuations. Thus, two residues that in some snapshots appear to be in close contact, may fall far apart for a brief period. To account both for the distance and for the fluctuations we calculated the geometric mean of the distance, a term that, in principle, is less sensitive to the contribution of extreme values and yields a smaller average value than the algebraic average. In order to identify the amino acids within helices D and E that have a direct interaction with the peptide, the strength of interaction was calculated according to Equations 5 and 6, and the normalized results are presented in Figure 5.

Analysis of the complex with the native peptide revealed that the nearest contact between the lid and the peptide is through Ser^595^, which is located on helix D. However, this residue is not the only one that is in close contact with the edge of the bound peptide, and Ala^592^ and Gln^596^ are also close enough (3–3.5 Å) that a water molecule could not squeeze between them (Figure 5, red bars).

On doubling the total charge of the peptide end (the N1R simulation), the pattern of interaction slightly changes, and the strongest interactions are now with Gln^585^, Gln^589^ and Ala^592^ (Figure 5, blue bars). Thus, it appears that the lid-peptide interaction is not mediated by specific residues, but with a region on the lid that serves as a contact domain. Substitution that eliminates the peptide charge (R2A—Figure 5, green bars), displays a significantly different pattern—the shortest geometric mean distance between the peptide and the lid is almost 6 Å long, which is spacious enough to allow at least one solvent molecule between the lid and the peptide.

### 2.5. The SBD^NR+^ (K577E) Simulation

An elegant procedure to monitor the *in vitro* activity of DnaK was introduced by Aponte *et al.* [14], who expressed various DnaK mutated variants at both domains of the protein. The chaperone activity was measured by the luminance of oxyluciferin through the co-expression of Luciferase with DnaK in bacteria. Using this technique, the researchers demonstrated that the K577E variant had activity which was ~4 folds lower as compared to the WT protein. Accordingly, we introduced the same replacement (K577E in the present sequence) and simulated the complex of the mutated SBD with the native peptide (simulation 7, Table 1). This charge reversal within the α-helices reduced the electrostatic potential operating within the lid sub domains, causing a structural rearrangement. The replacement of Lys with Glu at position 577 seemed to enhance the electrostatic attraction between the helices. The resulting structure of the DnaK^K577E^ was so stable, that its total MSD value throughout the whole simulation was less than 2 Å (data not shown).

**Figure 6:**
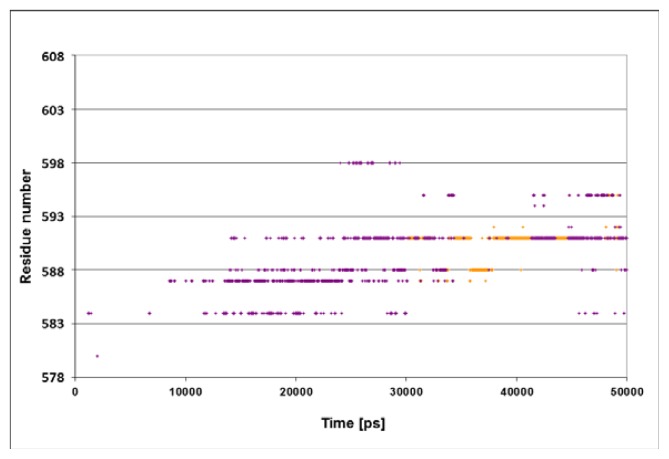
Spatiotemporal analysis of the interactions between residues on the lid, subjected to specific mutation (K577E) with the peptide. The dots describe the spatiotemporal analysis of the simulations of the complex of the mutated SBD (K577E) with the NR peptide. The color code for the distances is as in Figure 3.

The spatiotemporal analysis of the K577E simulation showed that there is no specific moiety on the lid that comes into contact with the peptide (Figure 6). Thus, we suggest that this mutation increased rigidity of the lid by anchoring the helices one to the other, prevents Ser^595^ from approaching the Asn^1^ moiety of the peptide. The correlation between the stiffening of the protein, as shown in our simulation, and the reduction in the *in vivo* activity measured experimentally (Aponte *et al.* [14]), provides an encouraging support to our suggested hypothesis that the interaction between the polar moiety on the lid (either Ser^595^ or other polar residue) and the N terminal moieties of the NR peptides is an essential step in the catalytic cycle of the DnaK chaperone.

### 2.6. Proximity between Residues on the Lid

**Figure 7:**
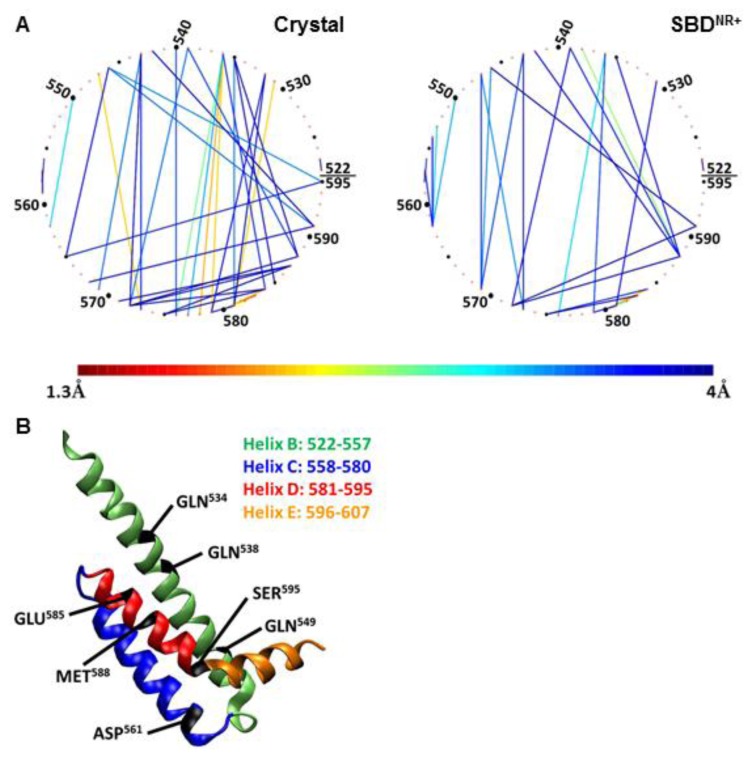
Interconnectivity network for helices B, C and D in SBD^NR+^ simulation. Frame **A** depicts the average distance between selected residues on helices B, C and D. Each residue is represented as a single dot on the circle and the distance to nearby residues is marked by a line whose color corresponds with the geometric mean of the minimal distance between the residues during the last 10 ns of the simulation time (see the color bar). The left panel represents the distances between the residues in the crystalline state of the protein, while the right panel represents the geometric mean of the minimal distance between two residues during the last 10 ns of the simulation. Only pairs for which the mean distance is less than 4 Å are shown. Frame **B** is a cartoon representation the structure of the lid with the residues marked in frame A. The helices are colored as in Figure 1. The limits of each helix are presented in the frame.

The simulations detailed in this study divert from the general notion that the lid is a uniform rigid body, which moves as a single block [11,26,30,34]. The interactions between the residues on the lid structure were evaluated by quantitation of the proximity factor between the lid residues. The analysis we carried out was focused on helices B, C and D, which are at the center of the lid, where most of the relative motions take place. The relative location of these helices was analyzed during two time frames, each 10 ns long; one at the initiation of the simulation, when the protein still maintains a low RMSD (Figure 2) and the shape is similar to that of the crystalline state, and one at the end of the simulations, where the RMSD had reached a new quasi stable level. The results of this analysis are presented in Figure 7 and Table 2.

To emphasize the nature of interaction between the residues on the lid (residues 522–595), they are arranged in Figure 7 as a virtual circle, and any pair of residues having a proximity factor of 4 Å (or less) are connected by a line colored according to the code given at the bottom.

Frame A in Figure 7 depicts the interactions between the residues located on helices B, C and D as calculated for the SBD^NR+^ simulation (right panel), and as compared to the starting point of the crystal structure.

This figure, as well as the data presented in Table 2, clearly shows that many of the contacts that existed in the crystal structure were lost during the simulation, and only few of them persisted. For example, the tight interactions in the crystal state of Arg^536^–Leu^576^ or Arg^547^–Glu^573^ were drawn further apart. Most of the continuous interactions along the simulation stabilized helix C in a close position relative to helix B. Another important interaction is between Met^588^ with helix B, which anchored helix D to helix B on one hand, but allowed the movement of Ser^595^ and helix E towards the peptide on the other.

**Figure 8:**
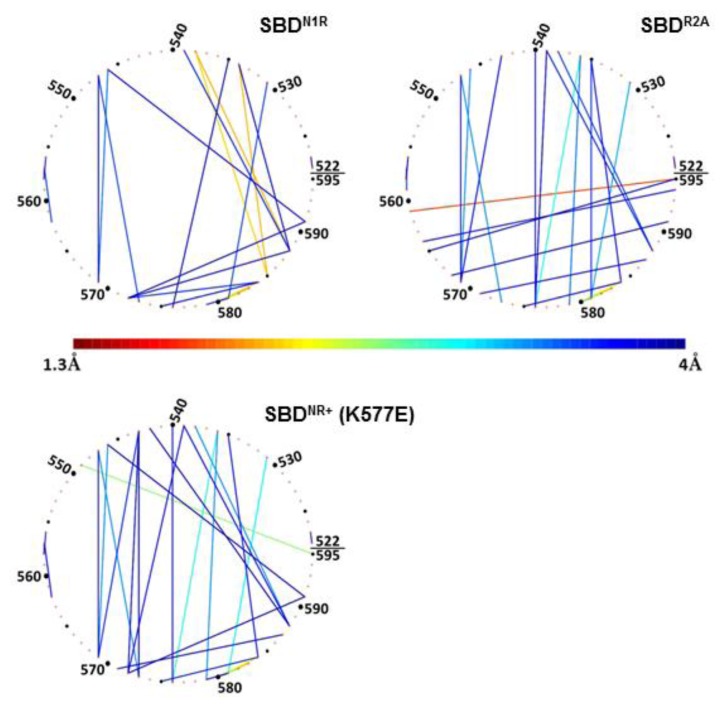
Interconnectivity network between helices B, C and D in SBD^N1R^, R2A and SBD^NR+^ (K577E) simulations. Each panel shows the geometric mean of the minimal distance between two residues during the last 10 ns of the simulation. The color of the connecting lines represents the mean distance according to the color bar. Only pairs for which the mean distance is less than 4 Å are shown.

The significance of the positive charge of the peptide on the compactness of the lid is emphasized in Figure 8 (N1R), depicting the analysis of simulation 5, where another positive charge was added to the peptide. In this complex, the residues making contact with the peptide are located on helix D (Figure 8, upper-left panel), but the pattern of inter-helices contacts differed from that of the WT complex; most of the interactions between helix B and helix C disappeared completely. At the same time, new strong interactions of less than 2 Å were formed between Gln^534^ and Gln^538^ on helix B and Glu^585^ and Met^588^ on helix D (as marked in Figure 8, and summarized in Table 2). This means that when the peptide had a double positive charge, helices C and D were rotated with respect to helix B, which in turn allowed a closer approach of helices D and E to the cavity in which the peptide was located.

In contrast with the pattern generated by a charged peptide, simulation of uncharged peptide (R2A) exhibited no tight contact between the tip of the peptide and any of the helices of the lid subdomain (Figure 4, frame B). The interconnectivity map in this case displayed a similar pattern to the WT simulation, with one considerable difference: Ser^595^, which normally had the most significant role in its contact with the peptide, was tightly bound to Asp^561^ (helix C).

Finally, once the lid was mutated (K577E) to a form where the *in vivo* activity of the protein was damaged, as was observed by Aponte *et al.* [14], the intra-lid contacts assumed a new pattern. This inversion of charge within the lid sub-domain, reinforced electrostatic attraction and reduced the average minimal distance between Glu^531^ on helix B and Lys^581^ on the loop between C and D helices, to ~3 Å (Figure 8, lower panel). We suggest that the stronger binding interaction between helix B and helices C and D stiffens the entire lid structure. The most pronounced outcome of this change is that Ser^595^, which in the WT complex was dedicated for interaction with the peptide, is now involved in an intensive interaction with Gln^549^ (helix B).

Despite the clear differences in the positions of the helices, several common connections can be identified from Table 2 (marked in bold in the table). We suggest that these contacts are essential to maintain the general integrity of the lid structure. Interestingly, not all of these contacts originate in the crystal structure of the SBD protein, which only emphasizes the importance of studying biological complexes as dynamic entities.

## 3. Discussion

DnaK is the bacterial protein representing the Hsp70 chaperone family that participates in energy driven protein (re)folding. During its functional cycle, the chaperone binds segments of unfolded proteins in its SBD. The affinity of the SBD to the protein alternate between the ATP- and ADP-loaded states of the NBD. The interaction between the SBD and the NBD is through a linked section which can transmit the stress caused by the nucleotide hydrolysis to the SBD, thus altering its affinity to the substrate.

In the present study we simulated the SBD component of the folding machinery, looking for spontaneous structural transitions that can be associated with the events controlling the affinity of the DnaK-peptide complex and the significance of the positively charged peptide for the proper function of the chaperone. For that purpose we used the crystalline SBD domain of DnaK with a bound NR peptide, (PDB ID: 1DKX) having a 2 Å resolution [20].

As emerges from our simulations, the solution structure of the SBD deviates from the crystalline one. During the first ~20 ns, the protein is still adhering to the initial structure, yet with time a new configuration is formed reaching a new quasi-stable state. In the present discussion we shall limit our evaluation to the observed transition, keeping in mind that further conformational changes, appearing at much later time, cannot be negated.

The analysis of the trajectories clearly revealed that the deviation from the crystalline state is mostly due to the deformation of the lid structure, while the β subdomain retained its original features. The lid structure, which is generally assumed to maintain a fixed orientation between helices C, D and E [21,22,26–28] appears to be flexible. When the peptide bound to the β subdomain bears positive charges, the lid alters its initial configuration and helices D and E reorient to protrude the junction between them toward the positive charges of the peptide. In case that the peptide is uncharged, no such deformation takes place. In case that the lid is mutated (K577E) so that the internal salt-bridge can rigidify the lid, the contact between the D and E junction with the peptide is prevented, together with loss of the chaperone activity of the mutant.

The structural transitions are coupled with the interactions between the lid’s residues that prevail in the crystalline state and their replacement by a new set of contacts. Apparently, the electrostatic potential which enables the motion of the lid enhances the formation of the contact, increasing the probability that the active form will be reached. Once the contact is made, other interactions (electrostatic and Lennard-Jones), both between the lid and the substrate and between moieties located on the inner helices (B, C and D) of the lid contribute to the stability of the structure.

The structural changes of the lid were investigated by Schlecht and coworkers who had measured the effect of immobilization of helices A and B on the activity of DnaK [22]. In their study helix A was linked by an artificial disulfide bond with the β subdomain, and the activity of the protein was found to be normal. On the other hand, when helix B was linked to the β subdomain, the rate of the reactions with either a protein or a short peptide was severely reduced. Moreover, the ability of the modified DnaK to refold luciferase was totally lost. The role of the relative motion of helix B was further emphasized by measuring the distance between the tip of helix B and the loop connecting β strands 5 and 6. In the absence of ATP, the two residues assumed two relative locations 12 and 20 Å apart, a distribution that was not affected by the presence of the substrate. Upon addition of ATP, the short distance conformation was lost. These observations are in accord with the present study, where the simulations were limited only to the SBD of the protein. In the absence of the specific interactions between the SBD with the NBD, both helices (A and B) practically retained their orientation with respect to the β subdomain. This feature was common both to the peptide-free SBD and in the presence of the peptide, even that the two residues of helix B (Gln^538^ and Gln^534^) form hydrogen bonds with the arginine moiety (Arg^2^) at the tip of the peptide. On measuring the distance between the same residues monitored by Schlecht, there was no significant difference between whether the DnaK was either loaded by the peptide or was in its apo state (16.5 ± 1.2 Å and 16.1 ± 0.9 Å). Apparently, the involvement of helix B in the overall catalytic cycle is associated with the ATP-dependent interaction between the NBD with the SBD, an event that is not covered in this study.

A protein in a solution samples a multitude of states and, under physiological conditions, can assume conformations that are far from the average one. If these low probability structures participate in the functional activity, the overall process must wait for their appearance, rendering them as the rate limiting step of the process. Thus, our postulation that the positive charge is necessary for the formation of contact between the lid and the peptide, does not imply that such contact may not happen in the absence of the positive charge; it only implies that the probability of having such a configuration will diminish, affecting the rate of all the processes where such conformational changes are a part of the overall reaction.

The enhanced probability of finding a contact between the lid and the peptide suggests that this relative motion of the helices is associated with the function of the protein. The observation of Aponte *et al.* [14] that the K577E mutation in the lid reduces the rate of the expression of luciferase in intact bacteria, is straightforwardly explained by the current simulations. The stiffening of the lid structure, reducing the ability of the lid to make contact with the peptide, must be tightly associated with the normal function of the system.

The contact between the peptide with the lid, stabilized by neutralization of local charges and Lennard-Jones interactions, allows the lid to transfer momentum to the peptide and facilitate its exit from the β sub domain to which it is tightly bound. Although the NBD driven “pulling” of the peptide by the lid has not yet been clarified, we can assume that during the catalytic cycle, the lid with the hooked peptide is dragged out of the crevice of the β sub domain. Once this happens, the stability of the lid-peptide contact will be weakened. At the high dielectric constant of a water exposed configuration the dissociation will be favored, the ionic screening will rapidly reduce the electrostatic attraction between the lid and the peptide and the solvation of the polar-charged residues will compensate for the loss of electrostatic potential. Thus, once the peptide is pulled out of the crevice by the lid, the peptide is prone to be released, bringing the chaperone activity to completion. In the case of partially folded proteins the observed interaction is difficult to reconcile due to steric considerations. However, the suggested mechanism is relevant for fully folded proteins or for peptides that are presented by Hsp70 chaperones [35].

## 4. Methods

### 4.1. DnaK SBD Structure

The coordinates of the SBD of the DnaK with a bound NR peptide, as determined by X-ray crystallography at a 2 Å resolution [20] were downloaded from the Protein Data Bank [36] (PDB ID: 1DKX). In some of the preformed simulations, we introduced point mutations to this crystallized structure using the SwissPdb Viewer software [37].

### 4.2. Molecular Dynamics Simulations Program

Standard MD simulations were carried out using the GROMACS 3.3.3 package [38–40] with the GROMOS96 force field, and a 53a6 parameter set [41]. The setup for simulation was as follows: As a preliminary step for the MD simulations, a dodecahedron box was built, with dimensions that extended at least 12 Å from the surface of the protein. The box was filled with water molecules using the spc216 model [42]. The charge of the protein was neutralized by the addition of counter-ions (Na^+^ and Cl^−^) in excess to increase the ionic strength up to 100 mM. The ionization of the residues was set so that that all carboxylates, including the C terminus of the SBD, were ionized, and all arginine and lysine side chains were positively charged. The N-termini of both the proteins and the peptide ligand were set to be uncharged (NH2) since, in vivo, they are covalently bound to the rest of their proteins.

MD simulations were carried out under NPT conditions of constant number of moles, pressure and temperature, using Berendsen’s coupling algorithm (*p* = 1 *b*ar; τ_p_ = 0.5 ps; *T* = 300 K; τ_T_ = 0.1 ps) [43]. The solvent and the solvated protein were thermally coupled as two separate groups. A 12 Å cutoff was used for the Van der Walls (VdW) interactions. The long-range electrostatic interactions were treated by the particle mesh Ewald (PME) [44]. The energy of each system was minimized using the steepest descent algorithm, followed by a conjugated gradient minimization. The resulting structures were simulated for 80 ps, while the protein’s position was restrained, in order to equilibrate the protein with the solvent. The system was then simulated for 1ns under no constrictions. After this equilibration, a production run was initiated with a 2 fs time-step. The initial velocities of all atoms in the system were randomly generated according to a Maxwell-Boltzmann distribution at 300 K.

### 4.3. The Contribution of Sub-Domains to the Total MSD of the Protein

For a protein made of well-defined domains which differ in their rigidity, the total MSD can be broken down to the relative values of each of the domains. The DnaK SBD is a good example of such a protein, with a relatively rigid β-sub-domain and more flexible α-helices. For this purpose we take the general expression for the Mean Square Deviation (*MSD*) of the whole protein (*MSD_whole_*), given by

Equation (1) and expand the right hand side as in Equation (2). In those equations $M_{whole}=\sum_{i=1}^{N}m_{i}$, and *r_i_*(*t*) is the position of atom *i* at time *t:*

(1) $${MSD}_{whole}(t_{1},t_{2})=\sum_{i=1}^{N}\frac{m_{i}{\left\||r_{i}(t_{1})-r_{i}(t_{2})\right\|}^{2}}{M_{whole}}$$

(2) $$\sum_{i=1}^{N}\frac{m_{i}{\left\|r_{i}(t_{1})-r_{i}(t_{2})\right\|}^{2}}{M_{whole}}=\sum_{domain\;1}\frac{m_{i}{\left\|r_{i}(t_{1})-r_{i}(t_{2})\right\|}^{2}}{M_{whole}}+\sum_{domain\;2}\frac{m_{i}{\left\|r_{i}(t_{1})-r_{i}(t_{2})\right\|}^{2}}{M_{whole}}+\cdots$$

where the total *MSD_whole_* is now presented as the sum of all domains. Algebraically, we used the Gromacs function g_rms and modulated it as given by Equation (3).

(3) $${MSD}_{domain}=\sum_{i=1}^{domain}\frac{m_{i}{\left\|r_{i}(t_{1})-r_{i}(t_{2})\right\|}^{2}}{M_{domain}}\times \frac{M_{domain}}{M_{whole}}$$

The advantage of these values is the additivity of the MSD function as in Equation (4).

(4) $${MSD}_{whole}=\sum_{i}^{n}{MSD}_{i}$$

where *n* is the total number of domains in the protein.

### 4.4. Residue-Residue Strength of Interaction

The purpose of this analysis is to identify persistent contacts between pairs of residues which last during a given time interval. The “proximity factor” for the interaction between a pair of residues is given in Equation (5) [45]. The term *P_ij_* is the geometric mean of the minimal distance between a pair of residues (*d_ij_*), as calculated over a predetermined time interval given by the number of simulation frames (*n*). The strength of the interaction between pair of residues (*S_ij_*) is defined as the inverse of *P_ij_* [Equation (6)].

(5) $$P_{ij}={\left(\prod_{t=1}^{n}d_{ij}(t)\right)}^{\frac{1}{n}}$$

(6) $$S_{ij}=P_{ij}^{-1}$$

This analysis was applied for phases during the simulation either close to the initial configuration where the structure still resembles the crystal structure, or towards the end of the simulation period where the structure appears to reach a stable configuration. In both cases the structures are flexible and the distance between the residues fluctuate with time. The “Proximity factor” is a measure to what extent the distance between two residues is stable, selecting for those pairs that maintain a close distance with small amplitude fluctuations. A cutoff value was used in the present study, eliminating pairs that do not contribute to the overall stability. When the proximity factor for a pair of residues exceeds 4 Å, these residues do not attract each other by any significant force based on electrostatic or Lennard-Jones potentials. The distance is longer than a stable hydrogen bond and the residues are sufficiently apart to accommodate a water molecule between them.

This analysis was used for two purposes. First, it was applied to identify the SBD residues which contact the NR peptide and its variants. Secondly, this technique was used to determine the interconnectivity network between the α-helices of the lid subdomain of the SBD.

### 4.5. Visual Presentation

All protein graphic presentations were generated by the VMD computer program [46].

## 5. Conclusions

The lid of Dnak is structurally flexible and it may play an active role in modulating the interaction between peptide and the SBD. The simulations revealed significant mobility within the “lid” structure, where the terminal two helices deviate from the crystal structure and establish contact with the positive charge at the tip of the NR. This contact is manifested only in the presence of electrostatic attraction. Mutations in the lid that render the protein inactive were shown to rigidify it, abolishing the interaction of helix D with the peptide.

The observed internal motions within the lid provide a molecular role for the function of this domain during the reaction cycle of Hsp 70 chaperons.

## Figures and Tables

**Table 1:** List of MD simulations.

	Simulation Type	Peptide	Simulation time (ns)
1	β-SBD^NR+^	NRLLLTG	50
2	β-SBD^NR−^	-	50
3	SBD^NR+^ (WT)	NRLLLTG	50
4	SBD^NR−^	-	50
5	SBD^N1R^	RRLLLTG	50
6	SBD^R2A^	NALLLTG	50
7	SBD^NR+^ (K577E)	NRLLLTG	50

**Table 2:** The geometric mean distance between residues on helices B, C and D of DnaK complex with the NR peptide. Only pairs for which the mean distance is not greater than 3.5 Å are shown. Common contacts, which appear in most of the simulations, are marked in blue.

	Residue 1	Residue 2	Distance(Å)
**Crystal**	**531**	**581**	**2.23**
	535	581	3.42
	536	578	2.06
	536	579	2.25
	**536**	**576**	**2.68**
	536	577	3.20
	539	572	3.39
	539	588	3.49
	540	576	3.52
	543	569	3.35
	546	595	3.25
	546	591	3.42
	**547**	**573**	**2.22**
	550	562	3.14

**WT**	**536**	**576**	**3.14**
	536	579	3.45
	**538**	**588**	**2.65**
	539	588	3.58
	543	569	3.49
	**546**	**569**	**3.27**
	**547**	**573**	**3.33**
	550	562	3.24
	553	562	3.09
	575	584	3.58

**SBD^N1R^**	**531**	**581**	**3.53**
	534	585	2.08
	538	585	2.23
	**538**	**588**	**2.11**
	**546**	**569**	**3.38**
	**547**	**573**	**3.50**
	557	562	3.54

**R2A**	**531**	**581**	**3.24**
	535	581	3.59
	**536**	**576**	**2.86**
	536	579	3.44
	**538**	**588**	**3.51**
	**546**	**569**	**3.34**
	**547**	**573**	**3.26**
	561	595	1.80

**SBDNR+(K577E)**	**531**	**581**	**3.04**
	**536**	**576**	**2.85**
	536	579	3.31
	**538**	**588**	**3.25**
	543	569	3.59
	**546**	**569**	**3.30**
	**547**	**573**	**3.26**
	549	595	2.59

## Abbreviations

LJ: Lennard-Jones
MD: Molecular Dynamics
PDB: Protein Data Bank
PLM: Palmitate
PME: Particle Mesh Ewald
RMSD: Root Mean Square Deviation
SPC: Simple Point Charge
VdW: Van der Waals
VMD: Visual Molecular Dynamics
mtHsp70: 70-kDa mitochondrial heat-shock protein
NR: the NRLLLTG hepta peptide

## References

[1] Burdon R.H. (1986). Heat shock and the heat shock proteins. Biochem. J.

[2] Jiang J., Prasad K., Lafer E.M., Sousa R. (2005). Structural basis of interdomain communication in the hsc70 chaperone. Mol. Cell.

[3] Montgomery D.L., Morimoto R.I., Gierasch L.M. (1999). Mutations in the substrate binding domain of the *Escherichia coli* 70 kda molecular chaperone, dnak, which alter substrate affinity or interdomain coupling. J. Mol. Biol.

[4] Zhang X.P., Elofsson A., Andreu D., Glaser E. (1999). Interaction of mitochondrial presequences with dnak and mitochondrial hsp70. J. Mol. Biol.

[5] Azem A., Oppliger W., Lustig A., Jenö P., Feifel B., Schatz G., Horst M. (1997). The mitochondrial hsp70 chaperone system. Effect of adenine nucleotides, peptide substrate, and mgrpe on the oligomeric state of mhsp70. J. Biol. Chem.

[6] Hermawan A., Chirico W.J. (1999). *N*-ethylmaleimide-modified hsp70 inhibits protein folding. Arch. Biochem. Biophys.

[7] Mapa K., Sikor M., Kudryavtsev V., Waegemann K., Kalinin S., Seidel C.A.M., Neupert W., Lamb D.C., Mokranjac D. (2010). The conformational dynamics of the mitochondrial hsp70 chaperone. Mol. Cell.

[8] Woo H.-J., Jiang J., Lafer E.M., Sousa R. (2009). Atp-induced conformational changes in hsp70: Molecular dynamics and experimental validation of an *in silico* predicted conformation. Biochemistry.

[9] Bertelsen E.B., Chang L., Gestwicki J.E., Zuiderweg E.R.P. (2009). Solution conformation of wild-type *E. coli* hsp70 (dnak) chaperone complexed with adp and substrate. Proc. Natl. Acad. Sci. USA.

[10] Bertelsen E.B., Zhou H., Lowry D.F., Flynn G.C., Dahlquist F.W. (1999). Topology and dynamics of the 10 kda c-terminal domain of dnak in solution. Protein Sci.

[11] Pellecchia M., Montgomery D.L., Stevens S.Y., Vander Kooi C.W., Feng H.P., Gierasch L.M., Zuiderweg E.R. (2000). Structural insights into substrate binding by the molecular chaperone dnak. Nat. Struct. Biol.

[12] Popp S., Packschies L., Radzwill N., Vogel K.P., Steinhoff H.-J., Reinstein J. (2005). Structural dynamics of the dnak-peptide complex. J. Mol. Biol.

[13] Wilbanks S.M., Chen L., Tsuruta H., Hodgson K.O., McKay D.B. (1995). Solution small-angle X-ray scattering study of the molecular chaperone hsc70 and its subfragments. Biochemistry.

[14] Aponte R.A., Zimmermann S., Reinstein J. (2010). Directed evolution of the dnak chaperone: Mutations in the lid domain result in enhanced chaperone activity. J. Mol. Biol.

[15] Smock R.G., Blackburn M.E., Gierasch L.M. (2011). The conserved, disordered c-terminus of dnak enhances its *in vitro* chaperone function and cellular survival upon stress. J. Biol. Chem.

[16] Greene M.K., Maskos K., Landry S.J. (1998). Role of the j-domain in the cooperation of hsp40 with hsp70. Proc. Natl. Acad. Sci. USA.

[17] Laufen T., Mayer M.P., Beisel C., Klostermeier D., Mogk A., Reinstein J., Bukau B. (1999). Mechanism of regulation of hsp70 chaperones by dnaj cochaperones. Proc. Natl. Acad. Sci. USA.

[18] Packschies L., Theyssen H., Buchberger A., Bukau B., Goody R.S., Reinstein J. (1997). Grpe accelerates nucleotide exchange of the molecular chaperone dnak with an associative displacement mechanism. Biochemistry.

[19] Suh W.C., Lu C.Z., Gross C.A. (1999). Structural features required for the interaction of the hsp70 molecular chaperone dnak with its cochaperone dnaj. J. Biol. Chem.

[20] Zhu X., Zhao X., Burkholder W.F., Gragerov A., Ogata C.M., Gottesman M.E., Hendrickson W.A. (1996). Structural analysis of substrate binding by the molecular chaperone dnak. Science.

[21] Marcinowski M., Höller M., Feige M.J., Baerend D., Lamb D.C., Buchner J. (2011). Substrate discrimination of the chaperone bip by autonomous and cochaperone-regulated conformational transitions. Nat. Struct. Mol. Biol.

[22] Schlecht R., Erbse A.H., Bukau B., Mayer M.P. (2011). Mechanics of hsp70 chaperones enables differential interaction with client proteins. Nat. Struct. Mol. Biol.

[23] Stevens S.Y., Cai S., Pellecchia M., Zuiderweg E.R.P. (2003). The solution structure of the bacterial hsp70 chaperone protein domain dnak(393–507) in complex with the peptide nrllltg. Protein Sci.

[24] Van Durme J., Maurer-Stroh S., Gallardo R., Wilkinson H., Rousseau F., Schymkowitz J. (2009). Accurate prediction of dnak-peptide binding via homology modelling and experimental data. PLoS Comput. Biol.

[25] Misra G., Ramachandran R. (2010). Exploring the positional importance of aromatic residues and lysine in the interactions of peptides with the plasmodium falciparum hsp70-1. Biochim. Biophys. Acta.

[26] Liebscher M., Roujeinikova A. (2009). Allosteric coupling between the lid and interdomain linker in dnak revealed by inhibitor binding studies. J. Bacteriol.

[27] Mayer M.P., Bukau B. (2005). Hsp70 chaperones: Cellular functions and molecular mechanism. Cell. Mol. Life Sci.

[28] Vogel M., Bukau B., Mayer M.P. (2006). Allosteric regulation of hsp70 chaperones by a proline switch. Mol. Cell.

[29] Chesnokova L.S., Slepenkov S.V., Protasevich I.I., Sehorn M.G., Brouillette C.G., Witt S.N. (2003). Deletion of dnak’s lid strengthens binding to the nucleotide exchange factor, grpe: A kinetic and thermodynamic analysis. Biochemistry.

[30] Moro F., Fernández-Sáiz V., Muga A. (2004). The lid subdomain of dnak is required for the stabilization of the substrate-binding site. J. Biol. Chem.

[31] Nicolai A., Senet P., Delarue P., Ripoll D.R. (2010). Human inducible hsp70: Structures, dynamics, and interdomain communication from all-atom molecular dynamics simulations. J. Chem. Theory Comput.

[32] Chiappori F., Merelli I., Colombo G., Milanesi L., Morra G. (2012). Molecular mechanism of allosteric communication in hsp70 revealed by molecular dynamics simulations. PLoS Comput. Biol.

[33] Marom M., Safonov R., Amram S., Avneon Y., Nachliel E., Gutman M., Zohary K., Azem A., Tsfadia Y. (2009). Interaction of the tim44 c-terminal domain with negatively charged phospholipids. Biochemistry.

[34] Buczynski G., Slepenkov S.V., Sehorn M.G., Witt S.N. (2001). Characterization of a lidless form of the molecular chaperone dnak: Deletion of the lid increases peptide on- and off-rate constants. J. Biol. Chem.

[35] Srivastava P. (2002). Roles of heat-shock proteins in innate and adaptive immunity. Nat. Rev. Immunol.

[36] Berman H.M., Westbrook J., Feng Z., Gilliland G., Bhat T.N., Weissig H., Shindyalov I.N., Bourne P.E. (2000). The protein data bank. Nucleic Acids Res.

[37] Guex N., Peitsch M.C. (1997). Swiss-model and the swiss-pdbviewer: An environment for comparative protein modeling. Electrophoresis.

[38] Berendsen H.J.C., van der Spoel D., van Drunen R. (1995). Gromacs: A message-passing parallel molecular dynamics implementation. Comput. Phys. Commun.

[39] Lindahl E., Hess B., van der Spoel D. (2001). Gromacs 3.0: A package for molecular simulation and trajectory analysis. J. Mol. Model.

[40] Van der Spoel D., Lindahl E., Hess B., Groenhof G., Mark A.E., Berendsen H.J.C. (2005). Gromacs: Fast, flexible, and free. J. Comput. Chem.

[41] Oostenbrink C., Villa A., Mark A.E., van Gunsteren W.F. (2004). A biomolecular force field based on the free enthalpy of hydration and solvation: The gromos force-field parameter sets 53a5 and 53a6. J. Comput. Chem.

[42] Berendsen H.J.C., Postma J.P.M., van Gunsteren W.F., Hermans J. (1981). Interaction models for water in relation to protein hydration. Intermol. Forces.

[43] Berendsen H.J.C., Postma J.P.M., van Gunsteren W.F., DiNola A., Haak J.R. (1984). Molecular dynamics with coupling to an external bath. J. Chem. Phys.

[44] Essmann U., Perera L., Berkowitz M.L., Darden T., Lee H., Pedersen L.G. (1995). A smooth particle mesh ewald method. J. Chem. Phys.

[45] Abramowitz M., Stegun I.A. (1964). Handbook of Mathematical Functions with Formulas, Graphs, and Mathematical Tables.

[46] Humphrey W., Dalke A., Schulten K. (1996). Vmd: Visual molecular dynamics. J. Mol. Graph.

